# Don’t blink: inattentional blindness in radiology report interpretation

**DOI:** 10.1259/bjro.20210030

**Published:** 2021-11-26

**Authors:** Malcolm M. Kates, Patrick O. Perche, Rebecca J. Beyth, David E. Winchester

**Affiliations:** ^1^ University of Florida College of Medicine, Gainesville, Florida; ^2^ Brookwood Baptist Health, Birmingham, Alabama; ^3^ North Florida/South Georgia GRECC, Malcom Randall VAMC, Medical Service, Gainesville, Florida; ^4^ Division of Internal Medicine, University of Florida College of Medicine, Gainesville, Florida; ^5^ Division of Cardiovascular Medicine, University of Florida College of Medicine, Gainesville, Florida

## Abstract

**Objectives:**

Medical errors attributable to inattentional blindness (IAB) may contribute to adverse patient outcomes. IAB has not been studied in the context of reviewing written radiological reports. This cross-sectional, deception-controlled study measures IAB of physicians towards an unexpected stimulus while interpreting written radiological reports.

**Methods:**

Physicians and residents from multiple fields were asked to interpret four radiology text reports. Embedded in one was an unexpected stimulus (either an abnormally placed medical exam finding or a non-medical quote from the popular television show *Doctor Who*). Primary outcomes were differences in detection rates for the two stimuli. Secondary outcomes were differences in detection rates based on level of training and specialty.

**Results:**

The unexpected stimulus was detected by 47.8% (*n* = 43) of participants; the non-medical stimulus was detected more often than the medical stimulus (75.0% vs  21.7%, odds ratio 10.8, 95% confidence interval 4.1–28.7; *p* < 0.0001). No differences in outcomes were observed between training levels or specialties.

**Conclusion:**

Only a minority of physicians successfully detected an unexpected stimulus while interpreting written radiological reports. They were more likely to detect an abnormal non-medical stimulus than a medical stimulus. Findings were independent of the level of training or field of medical practice.

**Advances in knowledge:**

This study is the first to show that IAB is indeed present among internal medicine, family medicine, and emergency medicine providers when interpreting written radiology reports.

## Introduction

Inattentional blindness (IAB) refers to the inability of an individual to perceive a stimulus in plain sight due to attention being focused on a different primary task. Substantial research has been done on IAB in fields such as psychology, but only recently has IAB been applied to the science of healthcare delivery.^1^ One of the primary hypotheses is that IAB exists as an adaptive mechanism that allows us to ignore distracting stimuli in order to maintain focus on the primary task at hand.^2^ This maintenance of focus at the cost of additional external stimuli could contribute to medical error in healthcare settings with potentially overwhelming numbers of stimuli.^3^

Several recent studies have shown how IAB can affect care delivery in emergency medicine, otolaryngology, nursing, and anesthesiology settings.^4–6^ Another recent study demonstrated how radiologists can be affected by IAB when interpreting CT images.^7^ This study expands this research by measuring the prevalence of IAB among non-radiologist physicians when interpreting written radiology reports.

## Methods

We conducted a cross-sectional, blinded deception study to establish the prevalence of physician IAB toward medical and non-medical stimuli while evaluating radiological reports. Subjects included residents and faculty physicians from departments of Internal Medicine (and subspecialties), Emergency Medicine, and Family Medicine. Subjects were recruited during teaching conferences for each of the aforementioned departments. A deceptive rationale for the study was given along with verbal informed consent.

Subjects received a packet, either Format A or Format B, of four typical radiology reports representing tests commonly seen and interpreted in each of these three fields with some level of pathology warranting further management (CT of head, CT of chest/abdomen, myocardial nuclear perfusion imaging, and CT coronary angiography) (Supplementary Texts 1 and 2). In the body of one of the four reports (not the conclusions), an unexpected written stimulus was inserted. Format A contained a sample of non-medical text from a popular television show (Moffat S. [2007], Blink, *Doctor Who*, BBC), while Format B contained a sample of medical text unrelated to the report (a normal eye examination) (Table 1). The order of the four cases did not differ between Format A and Format B and the unexpected stimulus was embedded in the same report in each. Participants were assigned to Format A *vs* Format B in a randomized 1:1 manner.

**Table 1.** Unexpected stimuli embedded in reports

Survey form	Stimulus type	Stimulus text in context
Format A	Medical	…The bony structures are free of lytic or blastic lesions. The pupils were equal, round, and reactive to light. Multilevel degenerative changes are seen involving the thoracolumbar spine…
Format B	Non-medical	…The bony structures are free of lytic or blastic lesions. Don’t blink, don’t even blink, blink and you’re dead. Multilevel degenerative changes are seen involving the thoracolumbar spine…

To simulate the environment of reading radiology reports without drawing attention to the unexpected stimulus, participants were given 12 minutes (3 minutes per case, timed) to read the reports and complete a brief written response form asking them to describe any abnormalities in the reports. In addition to detection of the errant text, clinicians were also asked to grade the degree of abnormality of the findings (normal, mild, moderate, or seriously abnormal) and provide a next step in management via free response (Supplementary Tables 1, 2 and Figure 2). Paper questionnaires were used for the ease of mass simultaneous participation and to allow anonymous data collection.

After completion of the survey, debriefing occurred for all participants as to the purpose of the study and the need for deception. Once informed, participants had the option to retain their questionnaire and exclude it from the study if they chose not to participate. An Institutional Review Board reviewed the study protocol and approved the use of deception (IRB201900242). The primary outcome of the study was to document the proportion of subjects who detected the unexpected stimulus and compare detection between the two formats via a χ^2^ test. In prior studies, between 17 and 35% of respondents successfully identified the subject of the IAB investigation.^4,7^ Based on a 30% rate of correct identification, and seeking to increase the identification rate to 60%, a sample of 96 would be needed to have power of 80% at α = 0.05. In pilot testing of the experiment, after initial blinded review, all study confidants were asked to look for the inserted text and readily identified it. Secondary outcomes were the differences in stimulus detection and degree of abnormality. Comparisons between medical specialties and training levels were made by χ^2^ and κ tests as appropriate. An α of <0.05 was considered statistically significant. Data were stored in a custom REDcap database and analysis was conducted using SPSS v. 21 (IBM, Armonk NY).^8^ The data sets generated during and/or analyzed during the current study are available from the corresponding author on request.

## Results

### Unexpected stimulus

Subjects consisted of 90 physicians (60 residents, 28 faculty, and 2 unknown). The plurality (*n* = 33) were from internal medicine, followed by emergency medicine (*n* = 24), family medicine (*n* = 18), internal medicine subspecialties (*n* = 12), and other (*n* = 3). Median number of years in practice for faculty was 19 (5–30 interquartile range). Format A was completed by 46 respondents; Format B by 44 respondents.

**Figure 1.**
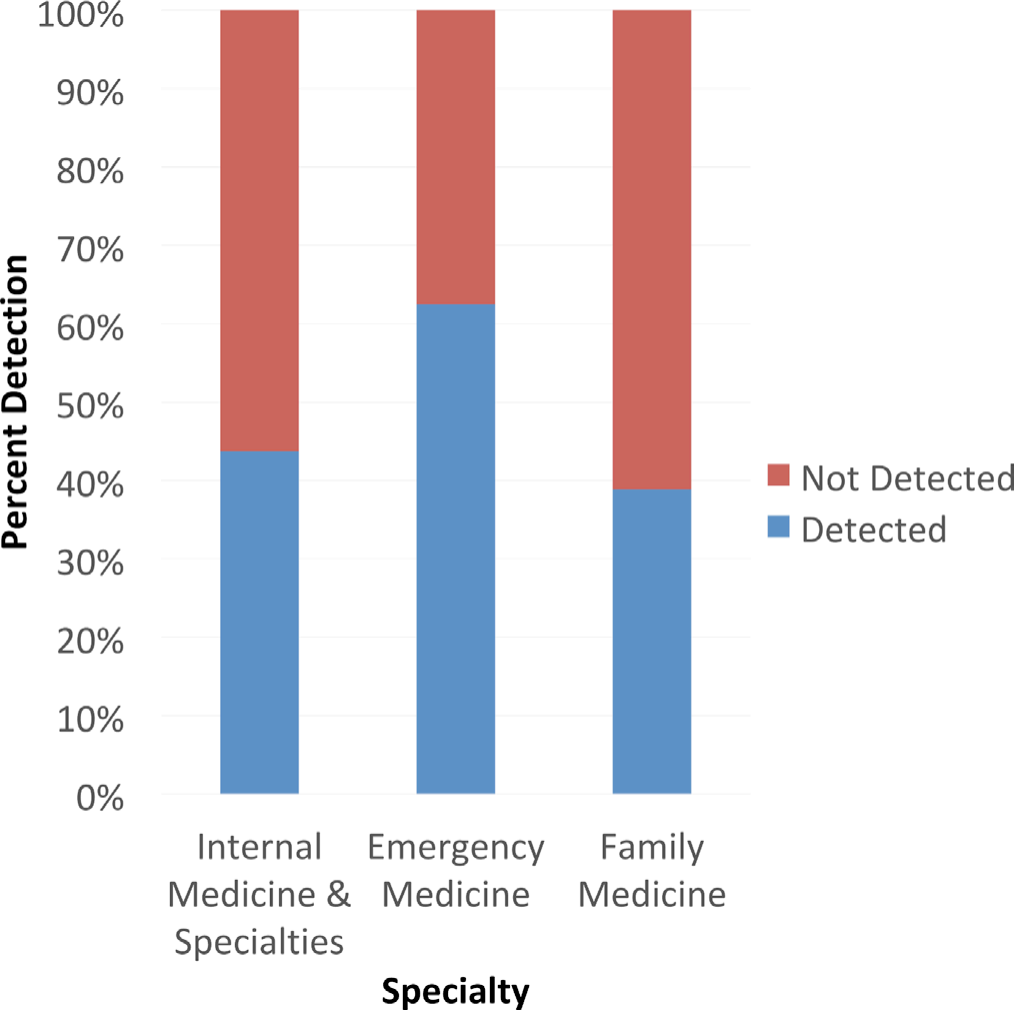
Distribution of prompt detection based on medical specialty. In this stacked bar graph, the distribution of prompt detection is stratified based on medical specialty (internal medicine, its subspecialties, and other are included in the first category). No significant differences in detection were observed by specialty (*p* = 0.11)

Overall, 47.8% (*n* = 43/90) subjects detected the unexpected stimulus in the report. Subjects were less likely to detect the medical stimulus (eye exam) (*n* = 10/43) than the non-medical stimulus (television show quote), (*n* = 33/43; 21.7 *vs* 75.0%, odds ratio 10.8, 95% confidence interval 4.1–28.7; *p* < 0.001,). Comparing stage of training, no difference in detection of the stimulus was observed between faculty (*n* = 17/43, 60.7%) and residents (*n* = 26/43, 43.3%; *p* = 0.13). No difference in detection of the stimulus was observed between medical specialties (internal medicine *n* = 21, 43.8% *vs* emergency medicine *n* = 15, 68.2% *vs* family medicine *n* = 7, 38.9%; *p* = 0.11) (Figure 1).

Most participants indicated observation of the prompts by underlining, circling, or restating the phrase in the response area. Some provided narrative comments on the prompts including: “most concerning is the non-sequitur buried in the comments”; “strange phrase: Don’t Blink”; “What??”; and “there is a typo circled above”. Only one participant mentioned that they would contact radiology regarding the prompt. One participant simply wrote in the margin “ha, ha, ha” with a drawing of a smiley face.

## Discussion

### Summary

Only a minority of physicians successfully detected an unexpected stimulus when interpreting a series of four radiology reports. Among those who did successfully detect the stimulus, they were significantly more likely to do so if the stimulus was non-medical in nature. This is consistent with understanding of how we process information and the concept of cognitive conspicuity, the “perceived relevance of the information”, such that a piece of information that stands out is more likely to capture our attention. However, IAB occurs when our past experiences and expectations divert our attention.^2^ Other factors that can influence IAB such as task interference and mental workload were less likely factors within our study as the subjects were not given a secondary diverting task or likely to be functioning “on autopilot” due to the setting of the study (during grand rounds presentation). No significant difference in detection rate was observed between medical specialties or training levels. This finding is not surprising, as IAB appears to occur to some degree among all people.^2^

IAB has previously been studied in the interpretation of radiological scans; however, we believe this is the first study to investigate IAB in physician interpretation of written radiology reports.^7^ Both our study and the prior radiological study asked subjects to detect a “nonsense” stimulus; one would not encounter a small gorilla on a CT scan or a TV quote in a radiological report in real-life. The phenomenon is consistent, however, with other studies on IAB in healthcare delivery that have studied the ability of subjects to detect clinically important information that, if not detected, could result in patient harm. One of the unique approaches with our methodology on IAB is using a deception design and a relatively minimal assessment tool. In contrast, most other studies have used complex or non-workplace designs that place the participants far afield from their daily work experience.^4,9^ While less robust, we believe our design better illustrates how likely IAB is to occur in a real-world setting.

### Strengths and limitations

The use of deception is essential to conducting an investigation of IAB. During the design and regulatory phase of the study, no one outside of the study team was made aware of the true purpose, except for the institutional review board officials. Enrollment occurred in three instances, although all were within 1 week to avoid subjects contaminating possible future subjects. While not specifically measured, we received no indication that any participants were aware of the true purpose of the study. Both self-deceptive enhancement (where the respondent unconsciously changes and truly believes their response) and impression management (where the respondent alters their response to improve their public perception) could contribute to over-reporting.^10^ Acting against these possibilities, the use of anonymous self-reporting in the study should have minimized temptation to over-report and thus reduced socially desirable reporting bias. Because physicians were asked what abnormalities they noted in each report, without specifically asking them if they noted the intentional abnormal stimulus, it is possible that not all those who detected the stimulus mentioned it in their response. This in turn could lead to an underestimate of the true detection rate. In the absence of a baseline stimulus detection rate, it is also possible that additional phenomena contributed in some way to physicians’ inability to detect either stimulus, though this was not tested. The study was conducted in an educational conference setting and we would not expect television show quotations to be accidentally included in a radiology report, therefore results could differ in real-world practice. Given that the conference setting actually minimizes external distraction, failure to detect the stimulus could be even greater in the real world. Taken together, various factors could independently lead to both over- and underestimation of the true detection rate and this simulated experiment may not perfectly encapsulate the role of IAB in a true clinical environment. Nonetheless, it provides new insight into the role of IAB in one distinct field within the broader medical landscape.

### Comparison with existing literature

As previously discussed (see Introduction), the presence of IAB and its contribution to medical error has been shown in several fields including critical care, anesthesiology, otolaryngology and nursing.^4–6^ This study was largely influenced by that of Drew et al studying the presence of IAB in the interpretation of lung CT images which found nearly 83% of trained radiologists failed to detect a gorilla superimposed into the CT image at hand.^7^ Recent findings from the same group (Williams et al) show that field-specific expertise alone does not protect against IAB.^11^ This finding is mirrored by the lack of correlation between stage of training and detection rates in this study. This study adds to the evidence for IAB as a potential source of error in other fields including emergency medicine and general practice. It also shows that IAB is not confined to the interpretation of visual images, but rather is present in text interpretation as well.

### Implications for research and/or practice

Having demonstrated that IAB can occur in a wide variety of clinical settings, the most important role for future research is to focus on strategies and interventions to reduce IAB among clinicians. Unlike some other biases and mental heuristics, prior research has suggested that knowledge and awareness of the IAB paradigm is not in and of itself protective.^9^ It may be necessary for other strategies to be developed in order to minimize the risk of errors due of IAB. Future generations of electronic health records may be designed to call attention to stimuli that might otherwise go unnoticed; others have suggested that electronic health records will incorporate artificial intelligence strategies to assist in decision-making. Future research should help clinicians develop individual- and system-based strategies to mitigate the influence of IAB in healthcare settings to minimize risks to patients.
